# Topical propranolol improves epistaxis in patients with hereditary hemorrhagic telangiectasia - a preliminary report

**DOI:** 10.1186/s40463-017-0235-x

**Published:** 2017-10-04

**Authors:** Meir Mei-Zahav, Hannah Blau, Elchanan Bruckheimer, Eyal Zur, Neta Goldschmidt

**Affiliations:** 1 The National HHT Center, Pulmonary Institute, Schneider Children’s Medical Center of Israel, 49202 Petach Tikva, Israel; 2 Cardiology Institutes, Schneider Children’s Medical Center of Israel, Petach Tikva, Israel; 3 Sackler Faculty of Medicine, Tel Aviv University, Tel Aviv, Israel; 4 Super-Pharm Professional - Central Compounding Pharmacy, Petach Tikva, Israel; 5 Department of Hematology, Hadassah-Hebrew University Medical Center, Jerusalem, Israel

**Keywords:** Hereditary hemorrhagic telangiectasia, Epistaxis, Propranolol, Anti-angiogenesis

## Abstract

**Background:**

Severe epistaxis is often difficult to control in patients with hereditary hemorrhagic telangiectasia (HHT). Propranolol has been shown to have antiangiogenic properties in vitro and in vivo and is commonly used to treat hemangiomas. We present our experience with topical nasal propranolol for the treatment of moderate to severe epistaxis in patients with HHT.

**Methods:**

Retrospective case series. Six patients with HHT were treated with 0.5 cm^3^ of 1.5% propranolol gel, applied to each nostril twice daily for at least 12 weeks. Outcome measures were epistaxis severity score (ESS), hemoglobin level, and number of blood transfusions prior to and while on treatment. Local and systemic side effects were recorded.

**Results:**

The mean duration of treatment was 30 ± 5.6 weeks. A significant improvement in the ESS was found in all patients, with a mean decrease from 6.4 ± 2.1 at treatment onset to 3.5 ± 1.7 at 12 weeks (*p* = 0.028). Hemoglobin level increased significantly from 8.4 ± 3.1 to 11.0 ± 1.8 g/dL at 12 weeks (*p* = 0.043). The mean number of blood transfusions decreased from 4.5 ± 4.9 before treatment to 2.5 ± 2.9 at 12 weeks and 0.3 ± 0.8 at 24 weeks, but the difference did not reach statistical significance (*p* = 0.109 for both). No significant side effects of treatment were recorded.

**Conclusions:**

These preliminary results suggest that topical propranolol may be effective for the treatment of epistaxis in patients with HHT. A prospective controlled trial is required to confirm our findings.

## Background

Hereditary hemorrhagic telangiectasia (HHT, Osler-Weber-Rendu disease) is characterized by the presence of abnormally dilated blood vessels, ranging from telangiectases in mucocutaneous tissues to large arteriovenous malformations in various organs including the lung, brain, and liver. The telangiectases tend to rupture, resulting in recurrent nasal or gastrointestinal bleeding [1]. In the majority of patients, the dysregulated angiogenesis and unbalanced generation of abnormal blood vessels are caused by mutations in the genes encoding endoglin and activin-like kinase 1 receptor (ACVLR1), components of the transforming growth factor-β receptor complex [1, 2]. Findings of increased levels of vascular endothelial growth factor (VEGF), a major angiogenic factor, in the blood and tissue of patients with HHT [3] suggest that it plays a role in the abnormal angiogenesis [2]. Therefore, VEGF may serve as a potential therapeutic target in HHT [4–6].

Propranolol is a nonselective beta blocker routinely used to treat hemangiomas in infants. It exerts an anti-angiogenic effect via apoptosis of endothelial cells and reduction of VEGF tissue expression [7]. Besides the few reports of topical timolol as a means of controlling epistaxis [8, 9], clinical data on propranolol as a therapeutic agent in HHT are scarce, and there are no reports of topical propranolol for epistaxis in this setting. The aim of the present case series was to describe our initial experience with the nasal application of propranolol gel to treat epistaxis in patients with HHT.

## Methods

### Patients

Between January 2014 and September 2015, patients with HHT who presented with moderate to severe epistaxis as evaluated by the Epistaxis Severity Score (ESS) [10] were treated with topical propranolol for a period of at least 12 weeks. As the treatment was novel and off label, we followed the patients carefully for safety and efficacy using a defined protocol. The inclusion criteria were: a clinical or genetic diagnosis of HHT; moderate to severe epistaxis (ESS- 4-10); failure of standard measures to control the bleeding including repeated cauterizations and laser applications, local or systemic tranexamic acid and in some patients, tamoxifen therapy; and no improvement with moisturizing gels administered locally for at least 8 weeks. The exclusion criteria were: congestive heart failure, baseline bradycardia, or any other contraindication for beta blocker treatment; on-going beta blocker therapy; any nasal surgery, laser treatment or cauterizations in the 3 months prior to treatment. The washout period after stopping beta blocker therapy was at least a month. Eligible patients were instructed to continue all other treatments with no change and to refrain from applying other local nasal agents.

Patients were instructed to visit their health care provider for blood pressure and heart rate measurements while receiving the propranolol gel therapy. Measurements were performed weekly the first 4 weeks and monthly thereafter.

For the present trial, the patients were seen in clinic at treatment initiation and every 3 months thereafter. The ESS was assessed at initiation of treatment as a score of 4–10 was a requirement for eligibility. In addition, since propranolol gel was a novel treatment we compared ESS and hemoglobin at onset of therapy and at 12 weeks to assess treatment efficacy.

### Medication

Treatment consisted of the application of 0.5 cm^3^ of 1.5% propranolol gel to each nostril twice daily for at least 12 weeks. The gel was prepared by a compounding pharmacy and contained propranolol hydrochloride 1.5% in isotonic and preserved hyperomellose gel. The gel was supplied with a plastic funnel-shaped guide attached to a syringe to avoid trauma to the nasal mucosa. Patients were instructed to insert the guide gently 0.5-1 cm into each nostril, to inject the gel into the nasal space and to inhale through the nostrils in order to spread the gel on the mucosa. Patients were instructed to do so in front of a mirror in order to apply the exact amount of gel prescribed.

The medication was prepared and supplied by one of the authors (EZ). A prescription for each patient was sent to the pharmacist monthly by 2 of the authors (NG and MM). As this was a new off-label therapy, patients reported use of the medication at each follow up visit, every 3 months.

Administration of the medication was approved by the hospital pharmaceutical committee. In accordance with the regulations for off-label use of a drug in Israel, all patients received a detailed explanation regarding the drug, and agreed to its off-label use.

### Outcome measures

The primary outcome measure was the change in ESS score. Secondary outcome measures were the change in hemoglobin and the change in the number of blood transfusions during 12 weeks after onset of therapy compared to the 12 weeks prior to therapy. The number of transfusions required in the 6 months following onset of therapy were also recorded when applicable.

### Statistical analysis

Continuous variables are summarized as mean and standard deviation. Wilcoxon signed-rank test was used to evaluate the difference between pre- and post-treatment ESS, hemoglobin levels, and transfusion requirements.

## Results

Nine patients were initially enrolled in the study. Of these, two had been scheduled for Young’s nasal closure prior to propranolol treatment, and despite an improvement after 4–8 weeks of treatment with propranolol they decided to undergo nasal closure. Since their ESS did not reflect 12 weeks of treatment, they were excluded from the analysis. A third patient was excluded because he had started systemic bevacizumab treatment for high cardiac output failure secondary to hepatic arteriovenous malformations.

The remaining six patients formed the study group. Table 1 summarizes their characteristics and disease course. The mean duration of treatment was 30 ± 5.6 weeks. At 12 weeks, there was a decrease in mean ESS from 6.4 ± 2.1 to 3.5 ± 1.7 (*p* = 0.028). The main improvements in the ESS parameters were a decrease in epistaxis frequency and duration. In three patients, the frequency of epistaxis episodes decreased from several times a day to less than once daily, and in two patients, the duration of the epistaxis episodes decreased from more than 30 min to less than 15 min. Three patients reported a change in epistaxis intensity from pouring and gushing to milder non-pouring bleeds. The mean hemoglobin level increased from 8.4 ± 3.1 g/dL at treatment initiation to 11 ± 1.8 g/dL at 12 weeks of treatment (*p* = 0.043).

**Table 1:** Clinical characteristics, treatment, and outcome of six patients with HHT treated with topical propranolol for epistaxis

Pt. no.	Demographics & HHT features			Topical propranolol treatment -- duration and outcome measures			
	Age/ gender (M/F)	HHT mutation	Other HHT features	Treatment duration (wks)	ESS before/ after 12 wks treatment	Hb (gr%) before/after 12 wks treatment	PC before treatment/after 12 wks/after 24 wks
1	72/M	Endoglin	PAVMs, GI bleed	48	7.3/1.5	7.4/10.9	10/3/0
2	55/F	Endoglin	HAVMs, GI bleed	12	4.4/1.9	12/12	–
3	68/F	ACVRL-1	PAVMs, HAVMs	38	10/5.9	5.4/9.9	9/6/0
4	41/M	ACVRL-1	PAVMs	34	4.4/2.5	12.5/14	–
5	37/F	ACVRL-1	None	26	6.2/4.2	6.1/10	–
6	76/F	Not done	HAVMs	26	6.4/4.9	7.1/9.1	8/6/2
*p*					0.028^a^	0.043^a^	0.109^a^ (12 weeks) 0.109^a^ (24 weeks)

^a^Wilcoxon signed-rank test

Abbreviations: *ESS* epistaxis severity score, *GI* gastrointestinal, *HAVMs* hepatic arteriovenous malformations, *Hb* hemoglobin, *PAVMs* pulmonary arteriovenous malformations, *PC* packed red cells

Patients with gastrointestinal bleeding (nos. 1 and 2, Table 1) did not experience a significant change in their gastrointestinal bleeds, suggesting the improvement in their hemoglobin levels was due to the decrease in nasal bleeding.

Blood transfusions were given as needed. Three patients required 8–10 transfusions of packed red cells each in the 12 weeks prior to the initiation of topical propranolol treatment (Table 1). The mean number of blood transfusions decreased from 4.5 ± 4.9 in the 12 weeks prior to treatment to 2.5 ± 2.9 at 12 weeks and 0.3 ± 0.8 at 24 weeks of treatment. The difference, however, did not reach statistical significance (*p* = 0.109 for both). On reviewing the data, we found that no transfusion had been given in the week prior to the hemoglobin measurement. However, as this study was retrospective, it was difficult to isolate the effect of any previous transfusion on the hemoglobin level.

All patients took iron supplements before and while on treatment. They were not prescribed any other treatment for epistaxis during the propranolol treatment period.

Patients were contacted monthly to monitor side effects and were reminded to record blood pressure and heart rate at their local clinic. Blood pressure and heart rate remained stable in all cases, and no significant side effects were reported. One patient complained of a burning sensation at treatment initiation which resolved within a few days.

## Discussion

Epistaxis in HHT can be severe, leading to secondary iron deficiency anemia and transfusion dependency [1], and it may not respond to standard measures. Treatment with different antiangiogenic agents has recently been proposed. Bevacizumab is so far the most studied [2, 4–6].

Topical propranolol is in widespread use for the treatment of infantile hemangiomas. Infantile hemangiomas share several characteristics with HHT, namely dysregulated angiogenesis and high levels of tissue VEGF [11]. Indeed, in vitro studies have reported that propranolol decreased the migration and angiogenesis of both normal as well as HHT endothelial cells [12]. Recently, oral propranolol was found to be effective in treating epistaxis in patients with HHT [13]**.** The present study is the first to show that epistaxis in HHT may also be relieved with propranolol gel. We observed a mean reduction of 3 ± 1.6 in the ESS after 12 weeks of treatment, which is considerably greater than the 0.71 change in ESS that was recently suggested to be clinically significant [14]. Other outcome measures were in concurrence with the decrease in ESS: hemoglobin level increased and the transfusion requirement decreased.

Propranolol is efficiently absorbed when applied to the nasal mucosa [15]. Systemic propranolol decreases heart rate and cardiac output resulting in decreased blood pressure. We did not observe these side effects in our patients, probably owing to the low daily cumulative dose of propranolol used (30 mg), which is considered sub-therapeutic for oral treatment of cardiovascular diseases in adults. This safety profile is in contrast to the significant side effects of other antiangiogenic medications used in patients with HHT [2, 4–6]. Furthermore, the cost of propranolol is very low.

Our study is limited by its retrospective nature. Although it is possible that the moisturizing effect of propranolol gel contributed to the reduction in epistaxis, our patients were instructed to use moisturizing gel for at least 2 months prior to propranolol treatment, and it failed to lead to significant improvement. An improvement in epistaxis parameters was also noted in the three patients who were excluded, but their period of treatment with propranolol was too short for analysis. These patients had either been scheduled for other treatments prior to propranolol initiation or were switched to systemic antiangiogenic agents to treat other symptoms, and propranolol was used as a bridge.

A controlled trial is required to further assess the safety and efficacy of propranolol gel for epistaxis in HHT.

## Conclusions

Our preliminary results suggest that nasal propranolol gel may have a place in the treatment of HHT-related epistaxis. A prospective randomized trial is planned to further assess the clinical efficacy and safety of topical propranolol for this purpose.

## Abbreviations

ESS: Epistaxis severity score
HHT: Hereditary hemorrhagic telangiectasia
VEGF: Vascular endothelial growth factor
